# SGA as a Risk Factor for Cerebral Palsy in Moderate to Late Preterm Infants: a System Review and Meta-analysis

**DOI:** 10.1038/srep38853

**Published:** 2016-12-13

**Authors:** Mengwen Zhao, Hongmei Dai, Yuanying Deng, Lingling Zhao

**Affiliations:** 1 Department of Pediatrics, the Third Xiangya Hospital of Central South University, Changsha, Hunan Province, China

## Abstract

Small for gestational age (SGA) is an established risk factor for cerebral palsy (CP) in term infants. However, there is conflicting data on the association between SGA and CP in moderate to late preterm infants. The aim of the article was to explore the relationship between SGA and CP in the moderate to late preterm infants and its strength by meta-analysis. We performed a system search in OVID (EMBASE and MEDLINE) and WANFANG from inception to May 2016. The study-specific risk estimates were pooled using the random-effect model. A total of seven studies were included in the meta-analysis, consisting of three cohort and four case-control studies. A statistically significant association was found between SGA and CP in moderate to late premature infants (OR: 2.34; 95% CI: 1.43–3.82). The association were higher in the several subgroups: 34–36 week gestational age (OR: 3.47; 95% CI: 1.29–9.31), SGA < 2SDs (OR: 3.48; 95% CI: 1.86–6.49), and malformation included in CP (OR: 3.00; 95% CI: 1.71–5.26). In moderate to late premature infants, SGA is a convenient and reliable predictor for CP. More studies are needed to explore the underlying mechanisms between SGA and CP association.

Cerebral palsy (CP), the most common motor disability in childhood resulting from a lesion caused by early insults to the developing brain, is a syndrome that has a serious impact on the life qualities of the affected children and their families. The prevalence of CP is approximately 1.8–3.5 per 1000 live births in developed countries^1^,^2^,^3^,^4^,^5^.However, there are no large-scale studies from developing countries, except for a study from China, which shows the prevalence of CP as 1.5 per 1000 live births^6^.

Improved as the perinatal and neonatal intensive cares are, the prevalence of CP remains relatively stable over the last several decades in developed countries^3^,^7^. It is suggested that perinatal disorders may not be the main cause of CP. Increasing evidence has demonstrated CP as a heterogeneous disease resulting from genetic factors, intrauterine triggers, and perinatal and neonatal diseases or their interaction effects^8^, such as maternal infection^9^,^10^, gestational age^5^,^11^,^12^,^13^, small for gestational age (SGA)^5^,^14^,^15^,^16^,^17^,^18^,^19^,^20^,^21^,^22^,^23^,^24^,^25^,^26^,^27^,^28^, birth defects^5^,^13^,^29^, placental conditions^5^,^10^,^30^, genetic mutations^31^,^32^, birth asphyxia^10^,^13^,^33^, and neonatal diseases^8^,^11^,^12^,^33^,^34^,^35^. Among them, gestational age and SGA, commonly referring to newborns whose birth weights are less than 10th percentile for the gestational age and considered to be a proxy for fetal growth restriction, have often been implicated as important risk factors for CP. In term infants, the association between SGA and CP is statistically significant^18^,^25^,^36^,^37^. However, in preterm infants, there is no consensus on the association between SGA and CP^5^,^14^,^33^,^38^,^39^,^40^,^41^. Gestational age appears to modify the association between CP and SGA.

Moderate to late premature infants are defined as those born between 32–36 weeks, which account for 84% of all premature births in the USA^42^. Moreover, the prevalence of CP is higher in this sub-category as compared to term group^5^. With the enhanced quality of perinatal management and health-care of premature newborns, concerns on the long-term neurodevelopment outcomes of those infants and opportunities for intervention were raised in the past few decades. SGA is a convenient and reliable predictor for neurodevelopment outcomes in term infants. However, in moderate to late premature infants, the inconsistency may result from small sample sizes and low statistical power, which could possibly show a large p value. The power for meta-analysis exceeds individual research. To better understand the association between SGA and CP so that more opportunities for intervention can be provided, we conducted a meta-analysis to assess SGA as a risk factor for CP in moderate to late premature infants.

## Methods

### Literature search

The electronic databases were searched from inception to May, 2016. A search was performed in OVID (EMBASE and MEDLINE) and WANFANG. For example, in MEDLINE, we used a combination of keywords, text words as well as word variants related to SGA, IUGR, CP, and searched medical subject headings: CP limited in epidemiology, etiology, prevention and control; fetal growth retardation and SGA limited in complications. A filter to identify case-control and cohort studies provided by BMJ Evidence Centre information specialists was used^43^. The research was also conducted in EMBASE using the similar terms. Furthermore, bibliography lists of relevant studies and previously published review articles were searched for potential studies. Only articles that were reviewed by peers were considered. The search strategy is described in the Appendix.

### Study selection

Inclusion criteria were as follows: (1) Case-control or cohort studies; (2) The study subjects included moderate or late premature infants; (3) The study aimed to explore the relationship between SGA and CP; (4) Relative risk (RR) or odds ratio (OR) and their corresponding 95% CIs of CP related to SGA were reported, or could be calculated from the data presented in the articles. We excluded animal studies, duplicates, case report studies, case series studies, and studies that evaluated the relationship between SGA and other neurodevelopmental conditions.

All titles and abstracts discussing the relationship between SGA and CP in premature infants were independently retrieved and screened by two authors (M.Z. and H.D.). If the titles or abstracts were considered as potentially relevant by both reviewers, the full-text of those identified studies were then assessed, with all references, author names, journal title, and funding sources concealed. Any disagreement was settled by discussion with the help of a third author (L.Z.).

### Data extraction

For articles meeting the criteria described above, the following data were independently extracted into standardized forms by two researchers (M.Z. and H.D.): publication year, birth year, malformation included in CP, the matched or adjusted variables, case-control or cohort study design, gestational age category, definition of SGA, study location, the first author and odds ratio with 95% confidence interval or crude data. When relative odds for the same study subjects were reported in multiple articles, the most recently published article was selected. When both crude and adjusted data were available in a study, only crude data were selected for the summary calculation since many included studies provided crude data and were not adjusted for the same confounders. The ORs were calculated with primary data presented in the articles and then pooled in the final meta-analysis, so as to maximize consistency of the individual risk estimates because most identified studies reported ORs rather than RRs. The OR can be a substitute for the RR when the prevalence of a disease is low (the prevalence of CP is 1.8–3.5 per 1000 live births). When inadequate information was provided for calculating OR, the original author was contacted for further information.

### Quality assessment

Two authors (M.Z. and H.D.) independently evaluated each included article using the Newcastle-Ottawa Scale on the basis of the qualities of the study group selection, comparability of study groups, and ascertainment of outcome assessment. The total quality scores ranged from 1 to 9, with higher scores indicating higher quality. Any disagreement was settled by discussion with the help of a third author (L.Z.).

### Statistical analysis

Statistical analysis was performed with R version 3.1.2 (‘meta’ package version 4.0–3). 95% CIs were calculated with crude data presented in the articles using the Woolf method. When zero cell was encountered, 0.5 was added into cell with corresponding 2*2 table. Since some studies provided multiple effect estimates on the basis of separate gestational age, an overall effect estimate was calculated from the available crude data using inverse variance method. The homogeneity was evaluated by Q test, preserved p = 0.1 used as heterogeneous criteria. A summary OR was calculated using randomized effect model, which takes weighted mean of each effect estimate. This model was chosen because of anticipated significant heterogeneity between studies on the basis of study methods and population. Funnel plot was used to detect potential publication bias. The trim and fill analysis was performed to assess the possible effect of publication bias on the meta-analysis. Subgroup analysis was performed using the predefined stratification according to study design, definition of SGA, gestational age, malformation included in CP, with p = 0.05 indicating statistical significance.

## Results

### Literature selection

**Figure 1:**
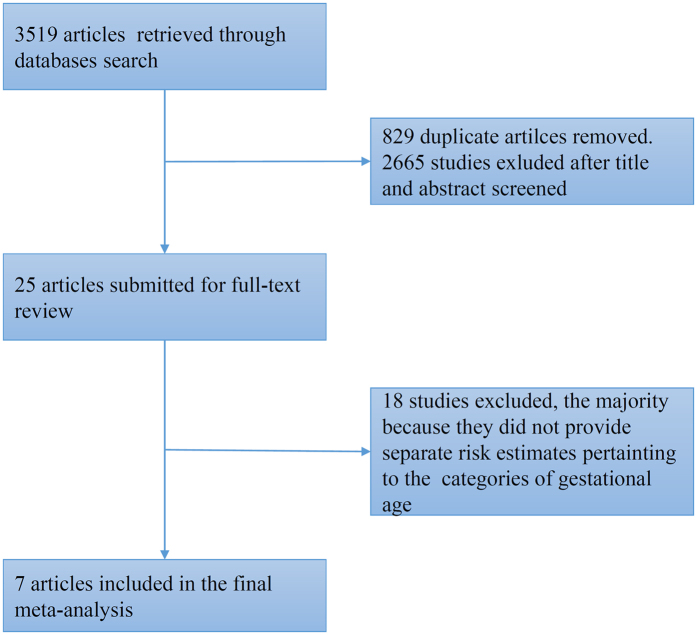
Flowchart of Literature Identification for Meta-analysis.

A total of 3519 articles were retrieved in accordance with the above search strategies. After read the titles and abstracts of all articles, 25 articles were selected for further review^5^,^14^,^15^,^16^,^18^,^19^,^30^,^33^,^38^,^39^,^40^,^41^,^44^,^45^,^46^,^47^,^48^,^49^,^50^,^51^,^52^,^53^,^54^,^55^,^56^. After assessing the full-text of the identified studies, seven articles were included in the meta-analysis^5^,^14^,^33^,^38^,^39^,^40^,^41^. The process for study selection and exclusion is detailed in Fig. 1.

### Characteristics of included studies

Details of included articles such as first author, location, birth year, study design, gestational age, SGA definition, sample size, outcome assessment, assessment of study quality, and matched or adjusted variables are presented in Table 1. Most studies were from developed countries (in Europe), with only one study from a developing country (China). The year of birth of the participants ranged from 1967 to 2008. The total number of participants included in this meta-analysis was 135650. Four studies defined SGA infants with birth weight less than 2SDs below the mean weight for gestational age, while the others defined SGA infants with birth weight less than 10th percentile for the gestational age. The potential confounders controlled in the included studies were different in different studies, with two studies presenting crude estimates. There was no significant association between SGA and CP in moderate to late premature infants in two studies^14^,^38^. Among seven studies, ORs ranged from 1.01 to 6.0. One article adjusted for the most confounders provided an adjusted OR (OR: 1.85; 95% CI: 1.25–2.75) in the 34–36 week gestational age and OR (OR: 1.10; 95% CI: 0.57–2.13) in the 32–33 week gestational age^5^. In most studies, the information on CP was extracted from registers, in which the diagnosis of CP was based on medical history, imaging data, and clinical multidisciplinary evaluations. Only two articles excluded participants with congenital malformation, which was an established risk factor for CP. The study quality scores ranged from 5 to 7. The median score was 6.

### Overall pooled effect

**Figure 2:**
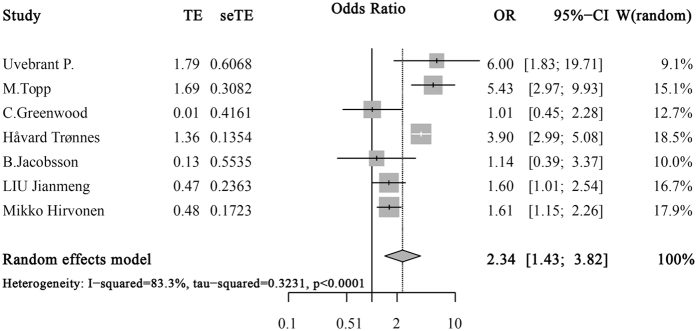
Meta-Analysis of SGA and CP in Moderate and Late Premature Infants. Squares represent study-specific estimates with their size reflecting the study-specific statistical weight; Horizontal lines represent 95% CIs; Diamond represents summary estimate with corresponding 95% CIs.

The overall pooled effect calculated from the seven articles demonstrated a statistically significant association between SGA and CP in moderate to late premature infants (OR: 2.34; 95% CI: 1.43–3.82), as was shown in Fig. 2. Significant heterogeneity was determined in the meta-analysis (p < 0.001), and a random effects-model was used.

### Subgroup analysis

The associations between SGA and CP in moderate to late premature infants in subgroup meta-analyses are shown in Table 2. The increased risk was more evident in several strata of study characteristics: using birth weight less than 2 SDs below the mean weight for gestational age to define SGA (OR: 3.48; 95% CI: 1.86–6.49), with gestational age limited in 34–36 weeks (OR: 3.47; 95% CI: 1.29–9.31), and malformation included in CP (OR: 3.00; 95% CI: 1.71–5.26). In most subgroup analyses, the SGA was a risk factor for CP and the associations were statistically significant. The risk of CP was higher in case-control studies than in cohort studies. However, case-control studies did not produce significant summary OR (OR: 2.48; 95% CI: 0.93–6.67), while cohort studies did (OR: 2.19; 95% CI: 1.15–4.18).

### Sensitivity and publication bias analysis

**Figure 3:**
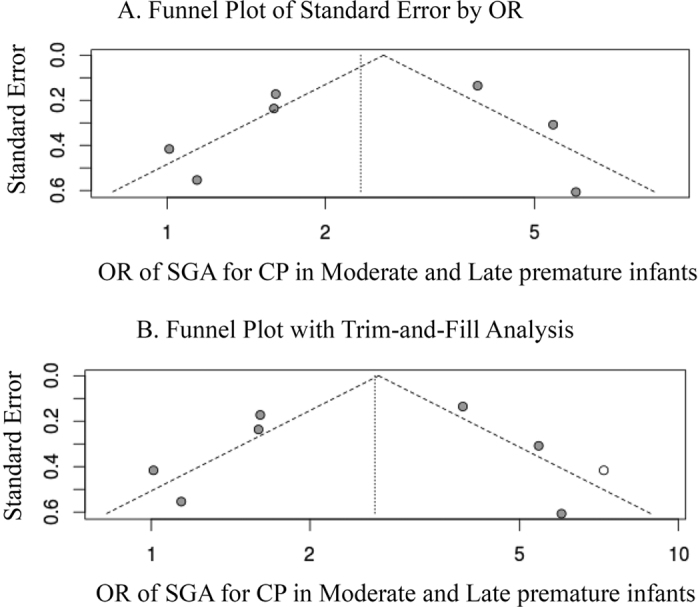
Funnel Plots for Detecting Publication Bias. Open circles suggest included studies, while the filled circles represent imputed studies identified from the trim and fill analysis.

The association between SGA and CP in moderate to late premature infants was determined by studies with different definitions of SGA and study design. However, removal of any study did not appreciably change the association. We performed a publication bias analysis by the funnel plot. The graph appeared to be asymmetrical, suggesting the existence of a publication bias (Fig. 3). The trim and fill analysis was performed and compared with the original results, which produced higher OR (OR: 2.65; 95% CI: 1.65–4.28; p < 0.001).

## Discussion

To our knowledge, no meta-analyses have previously explored the association between SGA and CP in moderate to late premature infants. Findings from the current research indicated that moderate to late premature infants with SGA had higher risk for CP. The finding was supported by five of seven studies included in this meta-analysis^5^,^33^,^39^,^40^,^41^.

From our meta-analysis evidence, SGA was a risk factor for CP in moderate to late premature infants. The higher risk for CP among children born with SGA is poorly understood. The association might be explained by several hypotheses: (1) Some factors result in both abnormal growth and brain damage; (2) Brain damage leads to abnormal growth or vice-versa; (3) Abnormal growth makes the newborns susceptible to negative perinatal events. According to the first two theories, SGA infants with CP are of prenatal origin. With the third, the perinatal events are the main sources, but with a latent prenatal factor. Current studies suggest that genetic and prenatal factors may be the primary causes for children born with SGA developing CP^24^,^25^. Besides, newborns with combined marked SGA and a major birth defect are at higher risk for CP^25^, as was confirmed by our subgroup analyses. Alternatively, some studies suggest that the risk of CP in children born with SGA is limited to those born with low Apgar scores^57^,^58^,^59^. Birth asphyxia may interact with poor fetal growth in the pathogenic pathway leading to CP^23^. Different pathophysiological mechanisms are probably associated with different intervention methods and long neurodevelopment outcomes. The potential causes should be actively explored in every infant with SGA.

In subgroup analyses, we showed that the pooled OR was not statistically significant in the subgroup, which defined SGA as <10th. Defining SGA as <10th is somewhat arbitrary because it may include not only pathological growth retardation but also constitutional smallness. This may be a reason why the relationship between CP and SGA has been described as paradoxical. Standard categorization of SGA is valuable for assessment of morbidity and mortality, better counseling, and potential early intervention. Our evidence supports that defining SGA as <2SDs may be a better cutoff for distinguishing pathological growth retardation from the population. When stratified by study design, the risk of CP was higher but not statistically significant in case-control study subgroup than in cohort study subgroup. This may be attributed to small sample sizes in case-control studies.

The significant association between SGA and CP appeared to be higher in 34–36 week gestational age in the subgroup analyses. It was apt to think that the extended effect of prenatal factors on fetus would put the fetus at an increased risk of developing CP. Some studies indicated that spontaneous preterm labor with SGA might be a fetal adaptive response^14^. However, we could not extract data on 32–33 week gestational age from the original articles and statistically significant association existed between SGA and CP in 32–36 week gestational age. More detailed gestational age classification will be needed in future studies.

In most studies, CP was defined as a group of non-progressive motor impair syndromes attributable to lesions or abnormalities of the developing fetal or infant brain. But the definition of CP used in the included studies differed in some respects. Some studies excluded children with clearly identified postnatal causes or malformation, while others did not. The age of CP diagnosis varied among the studies. Of all included studies, three made the diagnosis in children at least three years old, four without explicit age limitation. In our subgroup meta-analysis, the studies were classified by whether they excluded CP infants with malformation. The association between SGA and CP was weakened in subgroup with malformation excluded. Genetic mutations were suggested to be the cause of CP, and that a standard definition of CP is needed.

This meta-analysis has some limitations. First, we found significant heterogeneity across studies, which may result from differences in study design, sample size, definition of SGA, analysis strategies, and participants’ characteristics. But sensitivity analysis showed that the result was robust. The statistically significant associations were consistent in most subgroup meta-analyses. Second, matched or adjusted variables in most studies were different and two studies provided crude OR, which indicated the potential to wrongly attribute SGA to CP when other factors may contribute. However, a cohort study with the most adjusted confounders provided similar results^33^. Third, the funnel plot suggested a possible publication bias. The result from the trim and fill analysis was higher than the original outcomes. Finally, our pooled data contained few data of developing countries, thus lacking generalizability.

Among the children born with 32–36 week gestational age, strength of the association between SGA and CP was confirmed. Infants born in the 32–36 week gestational age with SGA showed up to 1.34 fold increase in the risk for CP (OR: 2.34; 95% CI: 1.43–3.82) as compared to infants with appropriate gestational age. In addition, the risk of developing CP was 3.48 (95% CI: 1.86–6.49) when SGA was defined as <2SDs. Accurate prediction of the risk of developing CP in moderate to late premature infants with SGA would be very valuable for early interventions to reduce the frequency of those adverse events. Additional research is needed to investigate the underlying causes between SGA and CP, which will likely improve clinical care, enable a more informed prognosis for those children and future pregnancies of their mothers, and contribute to a better understanding of the important relationship between SGA and CP.

## Additional Information

**How to cite this article**: Zhao, M. *et al*. SGA as a Risk Factor for Cerebral Palsy in Moderate to Late Preterm Infants: a System Review and Meta-analysis. *Sci. Rep.* **6**, 38853; doi: 10.1038/srep38853 (2016).

**Publisher's note:** Springer Nature remains neutral with regard to jurisdictional claims in published maps and institutional affiliations.

## Supplementary Material

Supplementary Appendix

## Figures and Tables

**Table 1:** Summary of Characteristics of Included Studies.

Source	Birth Year	Study Design	GA	SGA Definition	No. of Participants	No. of Cases	Outcome Assessment	Matched or adjusted variables	Quality assessment
Uvebrant P. *et al*. (Sweden)^41^	1967–1982	case-control	34–36	<−2SD	145	55	Medical records reviewed by physicians	Neonatal wards, GA, sex, birth date and type of delivery	6
Topp M. *et al*. (Denmark)^40^	1971–1982	case-control	34–36	<−2SD	1678	53	Cerebral Palsy Register of East Denmark	NA	5
Greenwood C. *et al*. (Britain)^38^	1984–1993	case-control	33–36	<−1SD	106	36	Oxford Register of Early Childhood Impairment	Neonatal ward, GA	6
Trønnes H. *et al*. (Norway)^5^	1967–2001	cohort	32–36	<−2SD	82702	NA	National Insurance Scheme of Norway	Pregnancy disorders, sociodemographic factors and year of birth	7
Jacobsson B. *et al*. (Sweden)^14^	1983–1990	case-control	34–36	<10th	73	24	The 1983–90 birth cohort of the cerebral palsy project in Western Sweden	Time of birth, gestational age, gender delivery ward	6
Liu J. *et al*. (China)^39^	1990–1997	cohort	32–36	<10th	4215	105	NA	NA	5
Hirvonen M. *et al*. (Finland)^33^	1991–2008	cohort	32–36	<−2SD	46731	385	Medical records and clinical multidisciplinary evaluation from the Hospital Discharge Register ICD-10 codes G80 to G83 in 1996 to 2008; ICD-9 codes 342 to 344 in 1991 to 1995	Time of birth, characteristics of mother, characteristics of delivery, newborn disorders	7

**Table 2:** Association between SGA and Cerebral Palsy in Moderate and Late.

Study Characteristics	No. of Studies	No. of Cases	P Value for Homogeneity	Relative Risk (95% Confidence Interval)
Study design				
Cohort	3		<0.001	2.19(1.15–4.18)
Case-control	4		=0.002	2.48(0.93–6.66)
Gestational age				
32–36	3		<0.001	1.89(1.04–3.43)
34–36	4		=0.04	3.47(1.29–9.31)
SGA definition				
<−2SD	4		<0.001	3.48(1.86–6.49)
<10th	3		=0.59	1.39(0.95–2.03)
Malformation included in CP				
No	2		=0.30	1.49(1.05–2.10)
Yes	5		=0.001	3.00(1.71–5.26)

Premature Infants Stratified by Study Characteristics.

## References

[b1] Yeargin-Allsopp M. . Prevalence of cerebral palsy in 8-year-old children in three areas of the United States in 2002: a multisite collaboration. Pediatrics 121, 547–554, doi: 10.1542/peds.2007-1270 (2008). 18310204

[b2] Stanley F. J. & Watson L. Trends in perinatal mortality and cerebral palsy in Western Australia, 1967 to 1985. BMJ (Clinical research ed.) 304, 1658–1663 (1992). 10.1136/bmj.304.6843.1658 PMC1882364 1633518

[b3] Hagberg B., Hagberg G., Beckung E. & Uvebrant P. Changing panorama of cerebral palsy in Sweden. VIII. Prevalence and origin in the birth year period 1991–94. Acta Paediatrica (Oslo, Norway: 1992) 90, 271–277 (2001). 11332166

[b4] Pharoah P. O., Cooke T., Johnson M. A., King R. & Mutch L. Epidemiology of cerebral palsy in England and Scotland, 1984–9. Archives of Disease in Childhood. Fetal and Neonatal Edition 79, F21–25 (1998). 9797620 10.1136/fn.79.1.f21 PMC1720819

[b5] Trønnes H., Wilcox A. J., Lie R. T., Markestad T. & Moster D. Risk of cerebral palsy in relation to pregnancy disorders and preterm birth: a national cohort study. Developmental Medicine and Child Neurology 56, 779–785, doi: 10.1111/dmcn.12430 (2014). 24621110 PMC4107088

[b6] J.-M. L. . Cerebral palsy and multiple births in China. Journal of Epidemiology (2000). 10.1093/ije/29.2.292 10817128

[b7] Slattery M. M. & Morrison J. J. Preterm delivery. Lancet (London, England) 360, 1489–1497, doi: 10.1016/S0140-6736(02)11476-0 (2002). 12433531

[b8] Wang L. W., Lin Y. C., Wang S. T., Yeh T. F. & Huang C. C. Hypoxic/ischemic and infectious events have cumulative effects on the risk of cerebral palsy in very-low-birth-weight preterm infants. Neonatology 106, 209–215 (2014). 25012626 10.1159/000362782

[b9] Neufeld M. D., Frigon C., Graham A. S. & Mueller B. A. Maternal infection and risk of cerebral palsy in term and preterm infants. Journal of Perinatology: Official Journal of the California Perinatal Association 25, 108–113, doi: 10.1038/sj.jp.7211219 (2005). 15538398

[b10] Freire G., Shevell M. & Oskoui M. Cerebral palsy: phenotypes and risk factors in term singletons born small for gestational age. European journal of paediatric neurology: EJPN: official journal of the European Paediatric Neurology Society 19, 218–225, doi: 10.1016/j.ejpn.2014.12.005 (2015). 25596065

[b11] Spinillo A. . Antenatal and delivery risk factors simultaneously associated with neonatal death and cerebral palsy in preterm infants. Early Human Development 48, 81–91 (1997). 9131309 10.1016/s0378-3782(96)01838-5

[b12] Beaino G. . Predictors of cerebral palsy in very preterm infants: the EPIPAGE prospective population-based cohort study. Developmental Medicine and Child Neurology 52, e119–125, doi: 10.1111/j.1469-8749.2010.03612.x (2010). 20163431

[b13] Sukhov A., Wu Y., Xing G., Smith L. H. & Gilbert W. M. Risk factors associated with cerebral palsy in preterm infants. The Journal of Maternal-Fetal & Neonatal Medicine: The Official Journal of the European Association of Perinatal Medicine, the Federation of Asia and Oceania Perinatal Societies, the International Society of Perinatal Obstetricians 25, 53–57, doi: 10.3109/14767058.2011.564689 (2012). 21463212

[b14] Jacobsson B. . Cerebral palsy and restricted growth status at birth: population-based case-control study. BJOG: an international journal of obstetrics and gynaecology 115, 1250–1255, doi: 10.1111/j.1471-0528.2008.01827.x (2008). 18715410

[b15] Gray P. H., Jones P. & O’Callaghan M. J. Maternal antecedents for cerebral palsy in extremely preterm babies: a case-control study. Developmental Medicine and Child Neurology 43, 580–585 (2001). 11570626 10.1017/s0012162201001074

[b16] Grobman W. A. . The association of cerebral palsy and death with small-for-gestational-age birthweight in preterm neonates by individualized and population-based percentiles. American Journal of Obstetrics and Gynecology 209, 340. e341–345, doi: 10.1016/j.ajog.2013.06.007 (2013). PMC3786044 23770470

[b17] Jarvis S. . Case gender and severity in cerebral palsy varies with intrauterine growth. Archives of Disease in Childhood 90, 474–479 (2005). 15851428 10.1136/adc.2004.052670 PMC1720399

[b18] Jarvis S. . Cerebral palsy and intrauterine growth in single births: European collaborative study. Lancet 362, 1106–1111, doi: 10.1016/S0140-6736(03)14466-2 (2003). 14550698

[b19] Drougia A. . Incidence and risk factors for cerebral palsy in infants with perinatal problems: a 15-year review. Early Human Development 83, 541–547, doi: 10.1016/j.earlhumdev.2006.10.004 (2007). 17188824

[b20] Lindqvist P. G. & Molin J. Does antenatal identification of small-for-gestational age fetuses significantly improve their outcome? Ultrasound in Obstetrics & Gynecology: The Official Journal of the International Society of Ultrasound in Obstetrics and Gynecology 25, 258–264, doi: 10.1002/uog.1806 (2005). 15717289

[b21] Stelmach T., Pisarev H. & Talvik T. Ante- and perinatal factors for cerebral palsy: case-control study in Estonia. Journal of Child Neurology 20, 654–660 (2005). 16225810 10.1177/08830738050200080401

[b22] Reid S. M. . Cerebral palsy and assisted reproductive technologies: a case-control study. Developmental Medicine and Child Neurology 52, e161–166, doi: 10.1111/j.1469-8749.2009.03556.x (2010). 20015250

[b23] Nielsen L. F. . Asphyxia-related risk factors and their timing in spastic cerebral palsy. BJOG: an international journal of obstetrics and gynaecology 115, 1518–1528, doi: 10.1111/j.1471-0528.2008.01896.x (2008). 19035988

[b24] Stoknes M. . Cerebral palsy and neonatal death in term singletons born small for gestational age. Pediatrics 130, e1629–1635, doi: 10.1542/peds.2012-0152 (2012). 23166338

[b25] Blair E. M. & Nelson K. B. Fetal growth restriction and risk of cerebral palsy in singletons born after at least 35 weeks’ gestation. American Journal of Obstetrics and Gynecology 212, 520. e521–527, doi: 10.1016/j.ajog.2014.10.1103 (2015). 25448521

[b26] Durkin M. S. . The role of socio-economic status and perinatal factors in racial disparities in the risk of cerebral palsy. Developmental Medicine and Child Neurology, doi: 10.1111/dmcn.12746 (2015). PMC4529795 25808915

[b27] Wu Y. W., Croen L. A., Shah S. J., Newman T. B. & Najjar D. V. Cerebral palsy in a term population: risk factors and neuroimaging findings. Pediatrics 118, 690–697, doi: 10.1542/peds.2006-0278 (2006). 16882824

[b28] Ahlin K. . Non-infectious risk factors for different types of cerebral palsy in term-born babies: a population-based, case-control study. BJOG: an international journal of obstetrics and gynaecology 120, 724–731, doi: 10.1111/1471-0528.12164 (2013). 23418811

[b29] Ancel P.-Y. . Cerebral palsy among very preterm children in relation to gestational age and neonatal ultrasound abnormalities: the EPIPAGE cohort study. Pediatrics 117, 828–835, doi: 10.1542/peds.2005-0091 (2006). 16510664

[b30] Murphy D. J., Sellers S., MacKenzie I. Z., Yudkin P. L. & Johnson A. M. Case-control study of antenatal and intrapartum risk factors for cerebral palsy in very preterm singleton babies. Lancet 346, 1449–1454 (1995). 7490990 10.1016/s0140-6736(95)92471-x

[b31] Hemminki K., Li X., Sundquist K. & Sundquist J. High familial risks for cerebral palsy implicate partial heritable aetiology. Paediatric and Perinatal Epidemiology 21, 235–241, doi: 10.1111/j.1365-3016.2007.00798.x (2007). 17439532

[b32] Tollånes M. C., Wilcox A. J., Lie R. T. & Moster D. Familial risk of cerebral palsy: population based cohort study. BMJ (Clinical research ed.) 349, g4294 (2014). 10.1136/bmj.g4294 PMC4099475 25028249

[b33] Hirvonen M. . Cerebral palsy among children born moderately and late preterm. Pediatrics 134, e1584–1593, doi: 10.1542/peds.2014-0945 (2014). 25422011

[b34] Tran U., Gray P. H. & O’Callaghan M. J. Neonatal antecedents for cerebral palsy in extremely preterm babies and interaction with maternal factors. Early Human Development 81, 555–561, doi: 10.1016/j.earlhumdev.2004.12.009 (2005). 15935933

[b35] Takahashi R. . Risk factors for cerebral palsy in preterm infants. Early Human Development 81, 545–553, doi: 10.1016/j.earlhumdev.2004.11.007 (2005). 15935932

[b36] S. M. . A systematic review of risk factors for cerebral palsy in children born at term in developed countries. Developmental Medicine and Child Neurology, doi: 10.1111/dmcn.12017 (2013). 23181910

[b37] MacLennan A. H., Thompson S. C. & Gecz J. Cerebral palsy: causes, pathways, and the role of genetic variants. American Journal of Obstetrics and Gynecology, doi: 10.1016/j.ajog.2015.05.034 (2015). 26003063

[b38] Greenwood C., Yudkin P., Sellers S., Impey L. & Doyle P. Why is there a modifying effect of gestational age on risk factors for cerebral palsy? Archives of Disease in Childhood. Fetal and Neonatal Edition 90, F141–146, doi: 10.1136/adc.2004.052860 (2005). 15724038 PMC1721863

[b39] Liu J., Li Z. & Lin Q. Intrauterine growth retardation and cerebral palsy. Chinese Journal of Preventive Medicine 35, 390–393, doi: 10.3760/j:issn:0253-9624.2001.06.009 (2001). 11840767

[b40] Topp M., Langhoff-Roos J., Uldall P. & Kristensen J. Intrauterine growth and gestational age in preterm infants with cerebral palsy. Early Human Development 44, 27–36 (1996). 8821893 10.1016/0378-3782(96)82791-5

[b41] Uvebrant P. & Hagberg G. Intrauterine growth in children with cerebral palsy. Acta Paediatrica (Oslo, Norway: 1992) 81, 407–412 (1992). 10.1111/j.1651-2227.1992.tb12259.x 1498507

[b42] Shapiro-Mendoza C. K. & Lackritz E. M. Epidemiology of late and moderate preterm birth. Seminars in Fetal & Neonatal Medicine 17, 120–125, doi: 10.1016/j.siny.2012.01.007 (2012). 22264582 PMC4544710

[b43] BMJ. Clinical Evidence. Study design search filters, http://clinicalevidence.bmj.com/x/set/static/ebm/learn/665076.html. Accessed May 6, 2015.

[b44] Guellec I. . Neurologic outcomes at school age in very preterm infants born with severe or mild growth restriction. Pediatrics 127, e883–891, doi: 10.1542/peds.2010-2442 (2011). 21382951

[b45] McElrath T. F. . Maternal antenatal complications and the risk of neonatal cerebral white matter damage and later cerebral palsy in children born at an extremely low gestational age. American Journal of Epidemiology 170, 819–828, doi: 10.1093/aje/kwp206 (2009). 19713285 PMC2765357

[b46] Costantine M. M., How H. Y., Coppage K., Maxwell R. A. & Sibai B. M. Does peripartum infection increase the incidence of cerebral palsy in extremely low birthweight infants? American Journal of Obstetrics and Gynecology 196, e6–8, doi: 10.1016/j.ajog.2007.01.009 (2007). 17466686

[b47] Thorngren-Jerneck K. & Herbst A. Perinatal factors associated with cerebral palsy in children born in Sweden. Obstetrics and Gynecology 108, 1499–1505, doi: 10.1097/01.AOG.0000247174.27979.6b (2006). 17138786

[b48] Spinillo A. . Rates of neonatal death and cerebral palsy associated with fetal growth restriction among very low birthweight infants. A temporal analysis. BJOG: an international journal of obstetrics and gynaecology 113, 775–780, doi: 10.1111/j.1471-0528.2006.00974.x (2006). 16753043

[b49] Glinianaia S. V. . Intrauterine growth and cerebral palsy in twins: a European multicenter study. Twin Research and Human Genetics: The Official Journal of the International Society for Twin Studies 9, 460–466, doi: 10.1375/183242706777591209 (2006). 16790158

[b50] Ehrenkranz R. A. . Growth in the neonatal intensive care unit influences neurodevelopmental and growth outcomes of extremely low birth weight infants. Pediatrics 117, 1253–1261, doi: 10.1542/peds.2005-1368 (2006). 16585322

[b51] Vohr B. R. . Center differences and outcomes of extremely low birth weight infants. Pediatrics 113, 781–789 (2004). 15060228 10.1542/peds.113.4.781

[b52] Matsuda Y. . Intrauterine infection, magnesium sulfate exposure and cerebral palsy in infants born between 26 and 30 weeks of gestation. European Journal of Obstetrics, Gynecology, and Reproductive Biology 91, 159–164 (2000). 10.1016/s0301-2115(99)00256-0 10869789

[b53] Kok J. H., den Ouden A. L., Verloove-Vanhorick S. P. & Brand R. Outcome of very preterm small for gestational age infants: the first nine years of life. British Journal of Obstetrics and Gynaecology 105, 162–168 (1998). 9501780 10.1111/j.1471-0528.1998.tb10046.x

[b54] Amin H., Singhal N. & Sauve R. S. Impact of intrauterine growth restriction on neurodevelopmental and growth outcomes in very low birthweight infants. Acta Paediatrica (Oslo, Norway: 1992) 86, 306–314 (1997). 10.1111/j.1651-2227.1997.tb08895.x 9099322

[b55] Veelken N., Stollhoff K. & Claussen M. Development and perinatal risk factors of very low-birth-weight infants. Small versus appropriate for gestational age. Neuropediatrics 23, 102–107, doi: 10.1055/s-2008-1071321 (1992). 1603283

[b56] Blair E. & Stanley F. Intrauterine growth and spastic cerebral palsy. I. Association with birth weight for gestational age. American Journal of Obstetrics and Gynecology 162, 229–237 (1990). 2301497 10.1016/0002-9378(90)90856-3

[b57] Westwood M., Kramer M. S., Munz D., Lovett J. M. & Watters G. V. Growth and development of full-term nonasphyxiated small-for-gestational-age newborns: follow-up through adolescence. Pediatrics 71, 376–382 (1983). 6828344

[b58] Berg A. T. Childhood neurological morbidity and its association with gestational age, intrauterine growth retardation and perinatal stress. Paediatric and Perinatal Epidemiology 2, 229–238 (1988). 2467264 10.1111/j.1365-3016.1988.tb00213.x

[b59] Berg A. T. Indices of fetal growth-retardation, perinatal hypoxia-related factors and childhood neurological morbidity. Early Human Development 19, 271–283 (1989). 2806156 10.1016/0378-3782(89)90062-5

