# Supplementary material for: SGA as a Risk Factor for Cerebral Palsy in Moderate to Late Preterm Infants: a System Review and Meta-analysis
Source: Sci Rep. 2016 Dec 13;6:38853. doi: 10.1038/srep38853 (PMC5153647; doi:10.1038/srep38853)
Supplement: Supplementary Appendix [file srep38853-s1.doc]

**Title:** SGA as a Risk Factor for Cerebral Palsy in Moderate to Late Preterm Infants: a System Review and Meta-analysis

**Authors and affiliations:** Mengwen Zhao^1^, Hongmei Dai^1^, Yuanying Deng^1^ and Lingling Zhao^1,*^

^1^ Department of Pediatrics, the Third Xiangya Hospital of Central South University, Changsha, Hunan Province, China

^*^Correspondence and requests for materials should be addressed to L.Z. (email:[llzhao2011@qq.com](mailto:llzhao2011@qq.com))

**Medline:**

exp cohort studies/

cohort$.tw.

controlled clinical trial.pt.

epidemiologic methods/

limit 4 to yr=1966-1989

exp case-control studies/

(case$ and control$).tw.

or/1-3,5-7

Cerebral Palsy/

"Neurodevelopmental outcome".mp.

"neuromotor outcome".mp.

"Cerebral Palsy".mp.

9 or 10 or 11 or 12

8 and 13

Fetal Growth Retardation/

Infant, Small for Gestational Age/

"Growth restriction".mp.

"Growth Retardation".mp.

"fetal growth restriction".mp.

"Fetal Growth Retardation".mp.

"intrauterine growth restriction".mp.

"intrauterine growth Retardation".mp.

"small for gestational age".mp.

"small for gestation".mp.

"impaired fetal growth".mp.

"small for date".mp.

"small for dates".mp.

"light for gestational age".mp.

"light for date".mp.

"light for dates".mp.

"birth weight".mp.

"perinatal factors".mp.

"risk factors".mp.

15 or 16 or 17 or 18 or 19 or 20 or 21 or 22 or 23 or 24 or 25 or 26 or 27 or 28 or 29 or 30 or 31 or 32 or 33

14 and 34

**EMBASE**

exp cohort analysis/

exp longitudinal study/

exp prospective study/

exp follow up/

cohort$.tw.

exp case control study/

(case$ and control$).tw.

or/1-7

cerebral palsy/ep, et, pc [Epidemiology, Etiology, Prevention]

"Neurodevelopmental outcome".mp.

"neuromotor outcome".mp.

"Cerebral Palsy".mp.

9 or 10 or 11 or 12

8 and 13

intrauterine growth retardation/

small for date infant/

"Growth restriction".mp.

"Growth Retardation".mp.

"fetal growth restriction".mp.

"fetal growth restriction".mp.

"intrauterine growth restriction".mp.

"intrauterine growth Retardation".mp.

"small for gestational age".mp.

"small for date infant".mp.

"small-for-gestational age".mp.

"small for gestation".mp.

"impaired fetal growth".mp.

"small for date".mp.

"small for dates".mp.

"light for gestational age".mp.

"light for date".mp.

"light for dates".mp.

"birth weight".mp.

"perinatal factors".mp.

"risk factors".mp.

15 or 16 or 17 or 18 or 19 or 20 or 21 or 22 or 23 or 24 or 25 or 26 or 27 or 28 or 29 or 30 or 31 or 32 or 33 or 34 or 35

14 and 36
